# Comparison of periodontal health in children with restored primary teeth using different restorative materials

**DOI:** 10.6026/973206300214388

**Published:** 2025-12-15

**Authors:** Aakash Vijay Patil, Nagaveni M. Sangavi, Manasi Kulkarni, Anuradha Jagtap, Sunny Tandon, Adhithi Prakash, Tanvi Hirani

**Affiliations:** 1 Department of Pedodontics and Preventive Dentistry, MGV'S KBH Dental College and Hospital, Nashik, Maharashtra, India; 2 Department of Pedodontics and Preventive Dentistry, ESIC Dental College and Hospital, Gulbarga, India; 3 Department of Pedodontics and Preventive Dentistry, Al Badar dental college and hospital, Gulbarga, Karnataka, India; 4 Department of Pedodontics and Preventive Dentistry, Kalka Dental College, Meerut, Uttar Pradesh, India; 5 Department of Pediatric and Preventive dentistry, School of Dental Sciences, Manav Rachna Dental College, Manav Rachna International Institute of Research and Studies, Faridabad, Haryana, India; 6 Department of Periodontology and Implantology, Karnavati School of Dentistry, Karnavati University, Gandhinagar, Gujarat, India

**Keywords:** Restorative materials, primary teeth, children

## Abstract

The quality of the restorative material used can have a significant impact on periodontal well-being in initial dentition though the comparative information is scarce. In this cross sectional study periodontal status of 240 children between 4-9 years of age fitted with restored primary molars with glass ionomer cement (GIC), composite resin, amalgam and stainless steel crowns (SSC) were compared and evaluated. Periodontal parameters such as gingivial index, plaque index, probing depth and gingivial recession were measured. It was proved that SSC caused much more inflammation and accumulation in gums than other materials (p<0.001) and GIC and composite resin did not differ in terms of periodontal outcome.

## Background:

Restoration of primary teeth by use of restorative procedures is important in the maintenance of the arch length, enabling mastication and the normal development of speech among pediatric patients [1]. Dental caries has been the most common chronic childhood illness with an estimated 60-90 percent of school going children worldwide suffering the disease and requiring regular restorative treatment [2]. Although the restoration of tooth structure and function has always remained the main object of the restorative dentistry, the effects of the restorative materials on the periodontal tissues have become more and more popular within the last few years [3]. The interaction between restorative materials and periodontal Clinical outcome is complex which entails the presence of variables like marginal adaptation, surface coarseness, possible plaque-gathering, or tissue biocompatibility [4]. Pediatric dentistry is now using different types of restorative materials, such as glass ionomer cement (GIC), composite resin, dental amalgam and stainless steel crowns (SSC) with different physical, chemical and biological characteristics [5]. The choice of the proper material to a restorative option must not be limited to durability and esthetics alone, but also the possible effects of the restorative material on the adjacent periodontal tissues [6]. The biocompatibility and tissue response of the various restorative types in primary teeth have been studied recently. Research has established that restoration overhangs are a significant problem to gingival inflammation in children [7]. In addition to this, it has been shown that surface properties of restorative materials have a direct effect on bacterial settlement and pandemic formation [8]. Glass ionomer cement has enjoyed extensive support as a component of pediatric restorations because of its release of the fluoride and chemical bonding to the tooth structure [9]. Stainless steel crowns on the other hand, though very durable in nature, were related to the increase in gingivial inflammation especially when the margin is sub gingivally extended [10]. The use of composite resins in pediatric dentistry has been popular as it has been found to be superior in esthetics and less preparation worry [11]. Research has discovered that well completed composite restorations have smooth surfaces which limit the retention of plaque [12]. Nevertheless, there is a lack of comparative information to assess the outcomes of periodontal outcomes with different restorative materials in the primary dentition. Rubio *et al.* (2025) addressed this gap by conducting a split-mouth clinical trial that directly compared periodontal health around stainless steel crowns with that of contralateral healthy primary molars in children, demonstrating significantly worse gingival and periodontal parameters in crowned teeth [13]. In spite of the existing research on individual restorative material, there is a lack of comparative studies examining periodontal health outcomes of commonly used pediatric restorative materials in one study. The majority of earlier studies have addressed the restoration longevity and clinical success as opposed to the reaction of the periodontal tissue [14]. Also, very little literature has measured a combination of various parameters of periodontal conditions in order to give a comprehensive picture of tissue health around the various types of restorative materials used in primary teeth. Therefore, it is of interest to compare periodontal health outcomes of the primary molars restored by four different restorative materials including glass ionomer cement, composite resin, amalgam and stainless steel crowns based on several periodontal parameters such as the gingival index, plaque index, probing depth and gingival recession.

## Materials and Methods:

## Sample size calculation:

Calculation of sample size was done with G * Power software version 3.1 with effect size of 0.25, alpha error of 0.05 and power of 0.80 to achieve significant differences between four groups. The minimum sample size was calculated to be 180 teeth. To offset possible dropouts and guarantee that there was appropriate coverage, 240 restored primary molars were factored in comprising 60 teeth in each group of restorative material.

## Study population:

One-on-one eligibility screening was done on children aged 4-9 years who were going to the pediatric dentistry outpatient department. The inclusion criteria included: systemically healthy children who had never used antibiotics within the last 3 months and had never used antibiotics; children who answered the question of cooperativeness; and restorations acceptable clinically with no apparent defects, secondary caries, or marginal breakdown.

## Exclusion criteria were:

Children with systemic diseases or conditions affecting periodontal health; children who received orthodontic treatment; teeth with apparent restoration failure, marginal failure or secondary caries; children with poor oral hygiene that did not respond to instruction; teeth with pathological mobility or periapical pathology and children who had received periodontal therapy within the previous 6 months.

## Study groups:

According to the restorative material employed, the restored primary molars were divided into four categories:

[1] Group 1: Glass ionomer cement restorations (n=60)

[2] Group 2: Composite resin restorations (n=60).

[3] Group 3: Amalgam restorations (n= 60)

[4] Group 4: Stainless steel crowns (n=60)

Clinical older adult physical examination and data gathering. The examinations of all the clinical activities occurred through the hands of one calibrated examiner who underwent training and calibration exercises with an intra-examiner kappa coefficient of 0.89. A Williams periodontal probe was used in the assessment of periodontal under sufficient illumination.

The periodontal parameters assessed were as follows, at four sites per tooth (mesio-buccal, mid-buccal, disto-buccal and lingual):

[1] Gingivality Index (GI): Evaluated based on the Loe and Silness criteria: 0 (healthy gingiva), 1(mild inflammation), 2 (moderate inflammation) and 3 (severe inflammation).

[2] Plaque Index (PI): Assessed according to Silness and Loe standards, 0 (no plaque), 1 (thin plaque film), 2 (moderate accumulation of plaque) and 3 (abundant accumulation of plaque).

[3] Probing Depth (PD): The four areas per tooth measured in millimeters to the gingival margin as far as the base of the sulcus

[4] Gingival Recession (GR): This is measured in millimeters between the cemento-enamel junction and the gingival margin. Demographic information such as age, gender and oral hygiene habits was present. The period after the restoration placement was recorded based on clinical recordings.

## Statistical analysis:

The data were keyed into the Microsoft Excel and analyzed using SPSS version 25.0. Descriptive statistics such as means, standard deviations, frequencies and percentages were determined. To determine normality of distribution the Shapiro-Wilk test was used. The continuous variables were compared in the four groups by one-way ANOVA and Tukeys test was followed by multiple comparison post-hoc test. Categorical variables were used to test through Chi-square. The correlation coefficient was used to determine the relationship between variables using Pearson correlation coefficient. A p-value below 0.05 was taken to be a significant figure.

## Results:

The final sample comprised 240 restored primary molars from 186 children (98 males and 88 females) with a mean age of 6.4 ± 1.3 years. The mean duration since restoration placement was 13.2 ± 4.8 months, with no significant difference among groups (p=0.312). Table 1 (see PDF) presents the demographic characteristics and baseline data of the study population. Table 2 (see PDF) demonstrates the comparison of periodontal parameters among the four restorative material groups. The mean gingival index was significantly higher in the SSC group (1.84 ± 0.62) compared to GIC (0.98 ± 0.43), composite resin (0.92 ± 0.38) and amalgam (1.26 ± 0.51) groups (p<0.001). Similarly, plaque index scores were highest in the SSC group (1.76 ± 0.58) and lowest in the composite resin group (0.87 ± 0.41), with statistically significant differences among all groups (p<0.001). Probing depth measurements revealed significantly greater values in the SSC group (2.34 ± 0.48 mm) compared to other materials (p<0.001). Gingival recession was observed more frequently around SSC (0.68 ± 0.42 mm) compared to tooth-colored restorations (p<0.001). The distribution of gingival health status based on gingival index scores is presented in Table 3 (see PDF). Healthy gingiva (GI=0) was most prevalent in the composite resin group (48.3%), followed by GIC (41.7%), amalgam (25.0%) and SSC (8.3%).

Severe gingival inflammation (GI=3) was exclusively observed in the SSC group (15.0%) and amalgam group (6.7%), while no instances were recorded in GIC or composite resin groups. Post-hoc analysis revealed that composite resin and GIC groups demonstrated statistically similar periodontal outcomes across all parameters (p>0.05), while both differed significantly from amalgam and SSC groups. The SSC group consistently exhibited the poorest periodontal health indicators across all measured parameters when compared to other restorative materials. Correlation analysis demonstrated a significant positive correlation between plaque index and gingival index scores (r=0.782, p<0.001), indicating that higher plaque accumulation was associated with increased gingival inflammation. Time since restoration placement showed weak positive correlation with gingival recession (r=0.234, p=0.003), suggesting progressive tissue changes over time.

## Discussion:

The current study was a comparative study that assessed and contrasted periodontal health outcomes of children treated with restored primary molars with four majorly used restorative materials. The results indicate that there are meaningful differences in periodontal parameters between the various restorative materials with stainless steel crowns having the most negative periodontal effects and tooth-colored materials, especially composite resin and glass ionomer cement having the best periodontal compatibility. The high level of gingivitis that was seen at stainless steel crowns in our study supports the earlier results reported by Randall *et al.* who found that the gingivitis index scores were higher by SSC than in intracoronal restorations [15]. Various reasons might cause this observation, such as placement of the subgingival margins, higher levels of retention of plaque and possible irritation of the tissue by the crown margins. The inconcusability of SSC commonly makes subgingival extension necessary to obtain satisfactory retention, which can be mechanically abrasive to the gingival structures and put in place conditions favoring the growth of plaque [16]. The similar periodontal results in the current study between composite resin and glass ionomer cement are comparable to the ones reported by Qudeimat *et al.* who have found similar gingival health indices of these two materials in primary teeth [17]. The two materials have the benefits of chemical or micromechanical bonding, which make it possible to establish conservative preparation designs and place supragingival margins. Moreover, the release of fluoride, which GIC exhibits, could also help it to provide such a good periodontal reaction by stopping bacterial colonization of the restoration margins. Several studies have reported the antimicrobial effects of materials that release fluoride and this may be the reason why there is less accumulation of plaque in the GIC group [9]. Superficial attributes of the restorative materials have a considerable effect on the accumulation of plaque and consequent gingival inflammation. The fact that we had found lower plaque index scores on composite resin groups can be explained by the fact that the surface of a modern composite material can be made smooth with the help of modern composite materials and correct finishing procedures [18]. A study by Beyth *et al.* has indicated that polished composite surfaces have lesser bacterial adhesion than amalgam or metal surfaces which confirms our observations [8]. A relatively rougher surface of the stainless steel crowns, along with margins and adaptation differences, were probably the factors that led to the fact that the accumulation of plaque in the SSC group increased significantly. Amalgam restorations showed middle periodontal results between tooth-colored and stainless steel crown. Although amalgam has dominated the field of pediatric dentistry because of its soundness and ease of application, marginal integrity and surface nature have been raised as a concern. The amalgam corrosion products are likely to cause gingival discoloration and local tissue response and this could be the reason as to why moderate inflammation of the gingiva is observed [4]. Nevertheless, modern clinical practice is less able to utilize these findings due to the persistent reduction in the use of amalgam in pediatric dentistry, especially primary teeth.

The greatly elevated probing depths and gingival recession that are witnessed around stainless steel crowns are of clinical concern. These results indicate that SSC can impair the periodontal attachment of the primary teeth which can have an impact on the developing periodontium. A study by Taran and Kaya (2018) reported that primary molars restored with stainless steel crowns demonstrated poorer gingival health and greater plaque accumulation compared with zirconia crowns and intact controls, rather than specifically documenting gingival recession linked to stainless steel crowns [10]. To reduce negative periodontal impact, clinicians must be keen to consider the position of margins and shape during the fabrication of SSC. New developments in SSC design such as pre-contoured crowns and better gingivism adaptation could be able to resolve some of these issues. The close positive relationship between the amount of the plaque and gingival inflammation that was recorded in our research supports the basic importance of teeth cleaning in periodontal health, irrespective of the restorative substance. This observation underscores the need of thorough oral hygiene education and care measures on children undergoing restorative therapy. The PCR pediatric should involve parent education and frequent professional prophylaxis as part of the restorative care [22]. Clinical implications of these findings indicate that in the event of choosing the restorative materials to use in primary teeth, clinicians should not just focus on the longevity of the restorations as well as the esthetics but also the possible periodontal effects. In cases where teeth that need restorations are close to the gingival tissues, tooth-colored restorations, including composite resin or GIC, might prove to be more periodontally compatible than prefabricated metal crowns. Nevertheless, it is necessary to resort to particular margin adaptation, correct choice of the crown and periodic monitoring of the periodontal status in cases when the SSC is clinically necessary because of the massive destruction of teeth. Limitations of the study are the cross-sectional design, which does not allow determining the cause-effect relationships and evaluating the changes in periodontal conditions over time. Longitudinal studies would also help to give crucial information about changes in periodontal progression surrounding varying restorative materials. Also, although some attempts were made to normalize examination conditions, the variability in the quality of restorations and a patient-specific factor might have affected results. It is also important to note that future investigations ought to inculcate standardized restoration protocols and extended follow-ups so as to holistically assess periodontal reactions to different types of restorative substances in primary dentition.

## Conclusion:

We show that composites resin and glass ionomer cement have better periodontal compatibility than amalgam and stainless steel crowns and should be used in preference as the restorative material of choice in periodontal health in primary dentition. Practitioners must place an emphasis on the materials that are related with positive periodontal outcomes during the restoration of primary teeth, which should be supported with the extensive maintenance programs of oral hygiene in order to promote the optimal periodontal health of pediatric patients in the long-term.
